# An Efficient Framework for EEG Analysis with Application to Hybrid Brain Computer Interfaces Based on Motor Imagery and P300

**DOI:** 10.1155/2017/9528097

**Published:** 2017-02-19

**Authors:** Jinyi Long, Jue Wang, Tianyou Yu

**Affiliations:** ^1^College of Information Science and Technology, Jinan University, Guangzhou 510632, China; ^2^School of Automation Science and Engineering, South China University of Technology and Guangzhou Key Laboratory of Brain Computer Interaction and Applications, Guangzhou 510640, China; ^3^Key Laboratory of Advanced Control and Optimization for Chemical Processes, Ministry of Education, East China University of Science and Technology, Shanghai 200237, China

## Abstract

The hybrid brain computer interface (BCI) based on motor imagery (MI) and P300 has been a preferred strategy aiming to improve the detection performance through combining the features of each. However, current methods used for combining these two modalities optimize them separately, which does not result in optimal performance. Here, we present an efficient framework to optimize them together by concatenating the features of MI and P300 in a block diagonal form. Then a linear classifier under a dual spectral norm regularizer is applied to the combined features. Under this framework, the hybrid features of MI and P300 can be learned, selected, and combined together directly. Experimental results on the data set of hybrid BCI based on MI and P300 are provided to illustrate competitive performance of the proposed method against other conventional methods. This provides an evidence that the method used here contributes to the discrimination performance of the brain state in hybrid BCI.

## 1. Introduction

Hybrid brain computer interfaces (BCIs) based on electroencephalogram (EEG) have attracted a great deal of attention because they can provide higher discriminant performance and more control commands compared to single model BCI [1–4]. In general, many research efforts have been focused on experiment paradigm design based on different BCI modalities to improve the discriminant performance [3–6]. However, in machine learning terms the methodology to analyze different patterns of BCI modalities is also important for discriminant performance improvement.

Signal analysis in BCI aims to predict the brain state of a user out of prescribed options [7, 8]. Many studies have focused on how to improve detection performance under the single modal BCI with different approaches. These approaches for data analysis have been applied in different steps such as feature extraction and selection (e.g., common spatial patter [9, 10]; independent component analysis coupled with heuristic frequency band selection [9]; band weighting [11, 12]) and classification (e.g., linear classifier [13–15], nonlinear classifier [14, 16, 17], and semisupervised learning [18, 19]). Furthermore, some efforts also try to develop a discriminant approach with a unified criterion for classifier coefficient (e.g., spatial filter and temporal filter) optimization from the training data [20–22].

Unlike the single modal BCI, there exist two or more brain patterns in the hybrid BCI (e.g., MI and P300). In machine learning terms, the challenge is that these patterns contain different order information in the signal [3]. For MI based BCI, the second-order information is used, while the first-order information is used for P300-based BCI. This leads to difficulty in the application of conventional statistical analysis to combine and learn brain patterns together. Many attempts to analyze the signal under the hybrid BCI are carried out though extracting the features from different modalities separately and then concatenating them to feed into some relative simple classifiers [2, 3]. However, these methods combine and learn the features indirectly which would lead to a nonoptimized resolution.

In this paper, we focus on the hybrid BCI paradigm based on our previous work, which includes MI tasks and P300 tasks. This indicates that the brain signal includes first-order and second-order information. To overcome the challenges described above, we propose using a discriminant approach that tries to combine and learn the hybrid features directly. The discriminant approach applied here has been proposed for single modality BCI by Tomioka and Müller [22]. The first-order information of the signal for P300 tasks and the second-order information of the signal for MI tasks are combined in a block diagonal form. These combined features can be selected and learned systematically with a linear classifier under dual spectral regulation. Our experimental results and data analysis demonstrate the efficiency of this discriminant approach.

## 2. Materials and Methods

### 2.1. Experiment and EEG Data Collection

A NuAmps device (Neuroscan) is used to measure scalp EEG signals for data acquisition. Each user wears an EEG cap (LT 37) that measures the signals from the electrodes. The EEG signals are referenced to the right ear. Two channels, “HEOG” and “VEOG,” representing eye movements are excluded (not shown here). The EEG used for processing is recorded from Ag-AgCl electrodes that are placed at the sites in the frontal, central, parietal, and occipital regions. The following 15 channels are included: “FC3,” “FCz,” “FC4,” “C3,” “Cz,” “C4,” “CP3,” “CPz,” “CP4,” “P3,” “Pz,” “P4,” “O1,” “Oz,” and “O2.” All impedances are kept below 5 kΩ. The EEG signals are amplified, sampled at 250 Hz, and bandpass filtered between 0.5 and 100 Hz.

**Figure 1:**
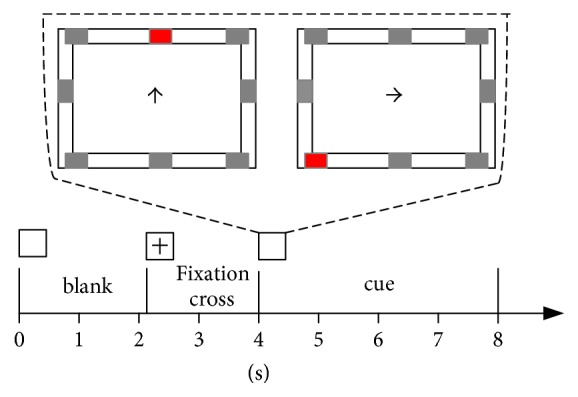
Paradigm for acquisition of data in a trial. At the beginning of the trial (0–2.25 s), the screen is blank. From 2.25 to 4 s a cross is shown onscreen to capture subject's visual attention. From 4 to 8 s, an arrow cue is provided. The subject is instructed to perform a mental task according to the following: right arrows cue right-hand motor imagery and up arrow cues attention to a specific button (center up button in this experiment).

**Figure 2:**
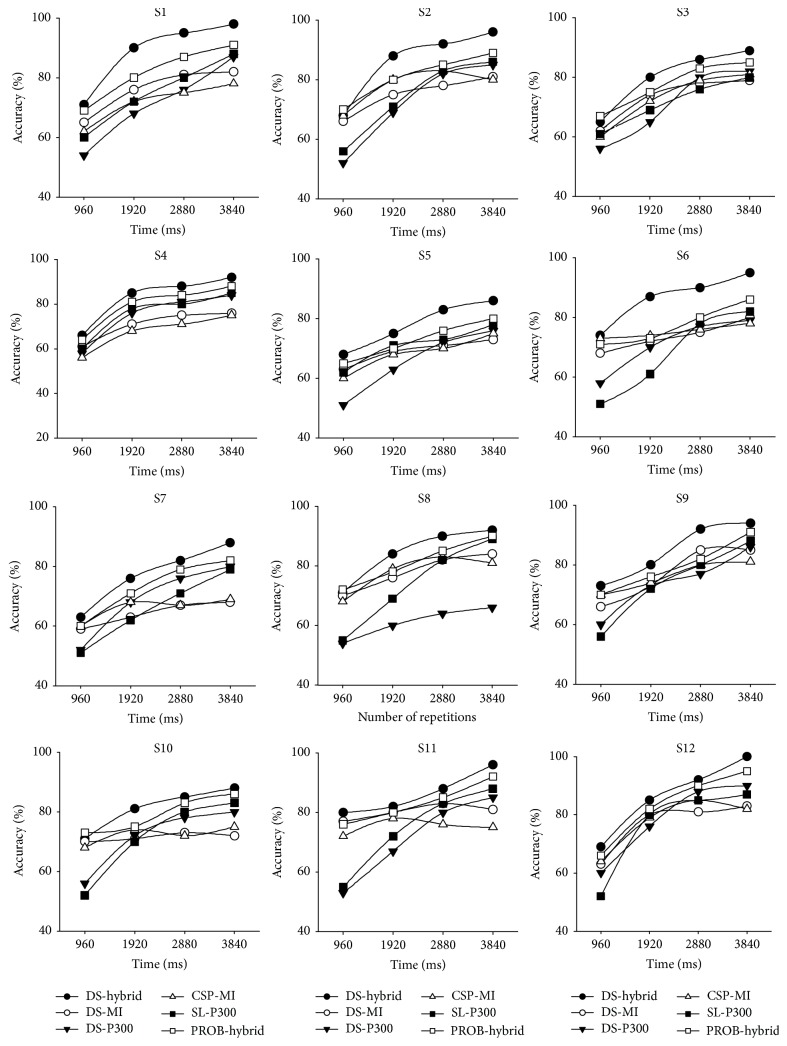
The individual accuracy across time.

In this experiment, the data was collected from twelve volunteers (10 males, 2 females) with ages in the range of 22–35 years. The graphic user interface used to combine P300 and MI is the same as described in our previous paper [2] and as shown in Figure 1. There are 8 flashing buttons around the screen. The trial design for data acquisition is shown in Figure 2. In the initial state (0–2.25 s) of each trial, the screen remains blank before a cross appears on the screen from 2.25 to 4 s to attract the subject's visual fixation. From 4 s to 8 s an up or right arrow cue is shown, and the subject is instructed to perform the P300 task or MI task (Table 1). The next trial begins after an interval of 4 s. During this interval the subjects were asked to relax. When the cue (i.e., up/right) appears the 8 buttons begin to alternately flash in a random order. Each button is intensified for 100 ms with a time interval of 120 ms between two consecutive button flashes. Thus, one round of button flashes occurs during a period of 960 ms, and each round is repeated 4 times in each trial. During the P300 task, subjects were instructed to focus on the up center button without any movement imagination, while during MI task, subjects were asked to perform right-hand imagery without any button attention. There are two sessions with each session comprised of 100 total trials (50 trials for each task). The first session is used to generate training data, and test data is derived from the second session.

### 2.2. Data Preprocessing and Pattern Extraction

This dataset involved two types of task: one related to P300 and the other corresponded to MI (Table 1). In the P300 task, the categories classified were the up center button attention or not (up or right arrow), while, in the MI task, the categories were the right-hand motor imagery and no motor imagery (up or right arrow). First, we introduce the data preprocessing procedure for these tasks separately below.

For the P300 task, the EEG signal is first bandpass filtered within the range of 0.1–20 Hz and then downsampled to 60 Hz. Next, the signal from a channel is segmented into epochs, each of which is from 0 to 600 ms after a flash of the button, specifically the up center button in this experiment. For each flash of a specific button in the *i*th trial, an epoch vector can be obtained by concatenating the data vectors derived from the 15 channels and denoted as *X*_P300_^(*i*,*l*)^ ∈ *R*^*T*×*C*^, where *T* = 37 and *C* = 15  (*l* = 1,…, 4). The feature vector in the *i*th trial *X*_P300_^(*i*)^ ∈ *R*^37  ×  15^ is obtained by averaging four epoch vectors corresponding to four repeats of specific button flashes and is assigned to a target *y* ∈ {+1, −1}. If the trial during training corresponds to attention to the specific button without motor imagery, then the label is set to +1. Otherwise, the label is −1. Then, we apply the spatial and temporal preprocessing matrices *P*^*s*^ and *P*^*t*^ to normalize each channel and time-point in *X*_P300_^(*i*)^ to unit variance as ${\overset{-}{X}}_{P300}^{\left(i\right)}=P^{t}X_{P300}^{\left(i\right)}P^{s}$. The *P*^*s*^ and *P*^*t*^ are defined as proposed in [22]. We also choose *P*^*s*^ = ∑^*s*−1/4^ and *P*^*t*^ = ∑^*t*−1/4^, where ∑^*s*^ = (1/*n*)∑_*i*=1_^*n*^cov(*X*_P300_^(*i*)^) and ∑^*t*^ = (1/*n*)∑_*i*=1_^*n*^cov(*X*_P300_^(*i*)^*T*^^) are covariance matrices in the spatial and temporal domain.

For the motor imagery task, EEG data were bandpass filtered within the range of 8–30 Hz and downsampled to 100 Hz. The bandpass filtered signal data *X*_MI_^(*i*)^ ∈ *R*^*C*×*T*^ for the *i*th trial was started during cue presentation and ended when the cue disappeared, where *C* = 15 and *T* = 400. The target of the *i*th trial is the same as the P300 task. Here, we used the pattern of the second-order covariance term for the motor imagery task. Similar with the normalization in the P300 task, this pattern is also normalized by applying a spatial whitening matrix ∑^*s*−1/2^ (i.e., Γ_MI_^(*i*)^ = ∑^*s*−1/2^cov(*X*_MI_^(*i*)^)∑^*s*−1/2^), where ∑^*s*^ = (1/*n*)∑_*i*=1_^*n*^cov(*X*_MI_^(*i*)^) is the covariance matrix in the spatial domain [22].

With the above extracted patterns of P300 ${\overset{-}{X}}_{P300}^{\left(i\right)}$ and motor imagery Γ_MI_^(*i*)^ for the *i*th trial, we can set *X*_P300,MI_^(*i*)^ as a block diagonal concatenation of both as shown below:

(1) $$\begin{array}{c} X_{P300,MI}^{\left(i\right)}=\left[\begin{array}{cc} \frac{1}{\xi_{1}}{\overset{-}{X}}_{P300}^{\left(i\right)} & \\ & \frac{1}{\xi_{2}}\Gamma_{MI}^{\left(i\right)} \end{array}\right], \end{array}$$

where *ξ*_1_ and *ξ*_2_ are the normalization factors used to standardize each feature to unit variance and defined as the square root of the total variance of each block element [23].

### 2.3. Linear Classification

The classifier used here is the linear function as shown below:

(2) $$\begin{array}{c} f_{\theta }\left(\;X_{P300,MI}^{\left(i\right)}\;\right)=\langle \;W,X_{P300,MI}^{\left(i\right)}\;\rangle +b, \end{array}$$

where *θ*≔(*W*, *b*), *W* is a matrix of some appropriate size, and *b* is a bias term. 〈*W*, *X*_P300,MI_^(*i*)^〉 = ∑_*j*,*k*_*W*(*j*, *k*)*X*_P300,MI_^(*i*)^(*j*, *k*) is the inner product between two matrices *X*_P300,MI_^(*i*)^ and *W* (*W*(*j*, *k*) denotes the (*j*, *k*) element of a matrix *W*). Denote *W* = ∑_*j*=1_^*J*^*b*_*j*_*w*_*j*_*w*_*j*_^*T*^, where *w*_*j*_ is the spatial filter and only the first several spatial filters are enough for good classification performance like a CSP based approach.

Before testing, parameters *θ* of the above linear classifier by training are obtained. With the training patterns *X*_P300,MI_^(*i*)^ and their corresponding true targets *y*_*i*_  (*i* = 1,…, *N*), the parameters can learn by solving the following constrained minimization problem with the dual spectral (DS) norm regularizer [22, 24, 25]:

(3) minθ∈Θ 1N∑i=1Nlog⁡1+e−yifθXP300,MIisubject to W∗≔∑jrδjW≤C,

where *δ*_*j*_(*W*) is the *j*th singular value of the weight matrix *W* and *r* is the rank of *W*. *C* is the hyperparameter that controls the complexity of the model and is selected by cross-validation with the training data set. For each subject, the *C* value was searched from 0.1 to 10 with a step of 0.2 and was set to the number with the best average performance after cross-validation.

Therefore, with the training parameters, we can predict the target of the pattern *X*_P300,MI_^(*t*)^ from the test data set as shown below:

(4) $$\begin{array}{c} y_{t}=\left\{\begin{array}{cc} +1 & \text{if }f_{\theta }\left(X_{P300,MI}^{\left(t\right)}\right)\geq 0,\\ -1 & \text{if }if_{\theta }\left(X_{P300,MI}^{\left(t\right)}\right)<0. \end{array}\right. \end{array}$$

As described above, we can see that the linear classifier can select and learn the features systematically under dual spectral regulation, in which the features are in a block diagonal form by combing the first-order information of the signal for P300 tasks and the second-order information of the signal for MI tasks. This framework can provide a way to optimize the features of MI and P300 together directly.

### 2.4. Validation Analysis

For comparison, we also performed the data analysis with the most used methods in BCI community. For the data analysis of MI task, we applied the common spatial patters (CSP) as the MI features and linear discriminant analysis (LDA) as the classifier (CSP-MI). While for the data analysis of P300 task, stepwise LDA was used as the classifier (SL-P300). To further prove the effect of our used method, we performed the classification using the PROB method [26], which we have presented previously [2, 3]. This method is used to combine the features of MI and P300 modalities. Specifically, two linear discriminant analysis (LDA) classifiers are trained using the MI feature vectors obtained by the CSP method and the P300 feature vectors with labels, respectively. Two scores for each trial's MI feature vector and P300 feature vector pair are computed using corresponding classifiers. If the average score is larger than 0, then the label is 1. Otherwise, the label is −1.

## 3. Results

Before performing the test, the regularization constant *C* by 10-fold cross-validation for each subject with the best performance was chosen as shown in Table 2. The classification performance obtained by the method proposed above using the chosen regularization constant is shown in Table 2 with an average accuracy of 92.8% (DS-hybrid). We also performed the classification with the MI and P300 separately as shown in Table 2. Their average individual classification accuracies are 79.6% (DS-MI) and 81.4% (DS-P300). The paired *t*-test showed that combining the MI and P300 resulted in better accuracy than that obtained by only MI (*p* < 0.001) or P300 (*p* < 0.001).

The classification performance with the standard algorithm for the data analysis with MI and P300 paradigms was 79.3% (CSP-MI) and 82.8% (SL-P300) as shown in Table 2 separately. The paired *t*-test statistical analysis showed that the classification accuracy obtained by DS-hybrid is better than that obtained by both CSP-MI (*p* < 0.001) and SL-P300 (*p* < 0.001) methods. In addition, the average classification accuracy is 87.6% (PROB-hybrid; Table 2). The paired *t*-test showed that the classification accuracy obtained by this method is also better than that obtained by the PROB method (*p* < 0.001). This result provides evidence of the efficiency of this method. We also performed 10-fold cross-validation with both sessions of data to replicate the results, providing further evidence of this method's efficiency. We also performed the classification for each repetition in the test set. As shown in Figure 2, classification accuracies after two repeats obtained using our method through combing MI and P300 (DS-hybrid) are more stable and better than that obtained with other methods. This indicates that better performance can be obtained with shorter time using our method.

**Figure 3:**
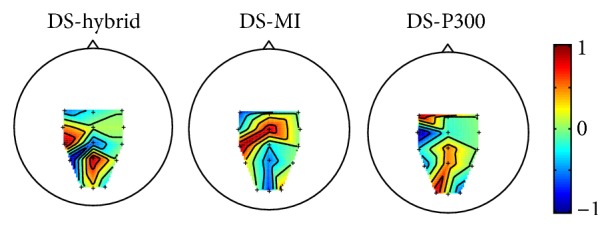
Scalp maps of channel weights for subject 1. All these mapping values are normalized separately to [−1 1].

Figure 3 shows the topographies of the channel weights (i.e., the mean of the first 15 of the first spatial filter for ${\overset{-}{X}}_{P300}^{\left(i\right)}$ and the last 15 values of the first spatial filter for Γ_MI_^(*i*)^ in (1) for DS-hybrid, the first row of CSP transformation matrix for MI paradigm and the classifier weights for P300 paradigm), obtained using the training dataset of S1. We can see that both the left motor cortex and occipital cortex contributed to the discrimination for DS-hybrid, while only left motor cortex for DS-MI and occipital cortex for DS-P300. This pattern of scalp map is consistent in all the subjects.

## 4. Conclusion

In this study, we propose to use a linear classifier with a dual spectral norm regularizer for multimodalities classification. Relative to the PROB or other conventional methods, this method can perform feature learning, feature selection, and feature combining directly through regularization other than indirect multistep. This method allows us to perform the feature learning jointly with the training of classifier in an optimization framework. Specially, this method can concatenate the features of MI and P300 in a block diagonal manner, allowing us to optimize them together through a more efficient method.

## Figures and Tables

**Table 1:** Experimental tasks.

Arrow cue	Task
Up	P300 task: focus on the up center button without any MI task
Right	MI task: right-hand imagery without any button attention

**Table 2:** Classification performance.

	DS-hybrid (%, *C*)	DS-MI (%, *C*)	DS-P300 (%, *C*)	CSP-MI (%)	SL-P300 (%)	PROB-hybrid (%)
S1	98 (0.9)	82 (1.3)	87 (2.3)	78	88	91
S2	96 (1.5)	81 (3.3)	85 (1.5)	85	87	85
S3	89 (5.7)	79 (0.3)	82 (0.9)	75	79	85
S4	92 (0.5)	76 (0.7)	84 (0.7)	80	76	88
S5	86 (2.9)	73 (2.5)	76 (1.3)	72	78	80
S6	95 (6.3)	80 (4.3)	79 (5.75)	81	83	86
S7	88 (4.1)	68 (1.5)	80 (6.3)	68	78	82
S8	92 (1.1)	84 (2.1)	66 (3.7)	82	80	90
S9	94 (2.5)	85 (3.3)	83 (1.7)	86	85	91
S10	88 (1.9)	76 (4.1)	80 (4.3)	74	82	86
S11	96 (0.3)	88 (1.5)	85 (5.7)	86	90	92
S12	100 (1.7)	83 (2.7)	90 (3.1)	85	88	95
Mean ± SD	92.8 ± 4.4	79.6 ± 5.6	81.4 ± 6.2	79.3 ± 5.9	82.8 ± 4.7	87.6 ± 4.3
